# Revealing Adenosine A_2A_-Dopamine D_2_ Receptor Heteromers in Parkinson’s Disease Post-Mortem Brain through a New AlphaScreen-Based Assay

**DOI:** 10.3390/ijms20143600

**Published:** 2019-07-23

**Authors:** Víctor Fernández-Dueñas, Maricel Gómez-Soler, Marta Valle-León, Masahiko Watanabe, Isidre Ferrer, Francisco Ciruela

**Affiliations:** 1 Unitat de Farmacologia, Departament de Patologia i Terapèutica Experimental, Facultat de Medicina i Ciències de la Salut, IDIBELL, Universitat de Barcelona, 08907 L’Hospitalet de Llobregat, Spain; 2 Institut de Neurociències, Universitat de Barcelona, 08035 Barcelona, Spain; 3 Department of Anatomy, Hokkaido University School of Medicine, 060-0818 Sapporo, Japan; 4 Institut de Neuropatologia, Servei d’Anatomia Patològica, IDIBELL-Hospital Universitari de Bellvitge, 08907 L’Hospitalet de Llobregat, Spain; 5 CIBERNED, Instituto Carlos III, 28035 Madrid, Spain; 6 Unitat de Anatomia Patològica, Departament de Patologia i Terapèutica Experimental, Facultat de Medicina i Ciències de la Salut, IDIBELL, Universitat de Barcelona, 08907 L’Hospitalet de Llobregat, Spain

**Keywords:** AlphaScreen, GPCR oligomerization, Parkinson’s disease, Adenosine A_2A_ receptor, Dopamine D_2_ receptor

## Abstract

**Background**: Several biophysical techniques have been successfully implemented to detect G protein-coupled receptors (GPCRs) heteromerization. Although these approaches have made it possible to ascertain the presence of GPCR heteromers in animal models of disease, no success has been accomplished in pathological human post-mortem brains. The AlphaScreen technology has been consistently used to quantify small analyte accumulation or depletion, bimolecular interactions, and post-translational modifications. The high signal-to-background, dynamic range and sensitivity exhibited by this technology support that it may be suitable to detect GPCR heteromers even under non-optimal conditions. **Methods**: Here, we describe the development of a new AlphaScreen assay to detect GPCR oligomers in human post-mortem brain. **Results**: Adenosine A2A-dopamine D2 receptor (A2AR/D2R) heteromer formation was monitored in caudate from healthy and Parkinson’s disease (PD) subjects. The approach was first validated using striatal membranes from wild type and A2AR deficient mice. Secondly, we took advantage of the 6-hydroxydopamine hemiparkinsonian rat model to validate previous results. In addition, finally, A2AR/D2R heteromer formation was assessed in caudate membranes from human post-mortem brains. Importantly, our preliminary results revealed an increase in A2AR/D2R heteromer formation in PD brains. **Conclusions**: The new AlphaScreen assay allowed assessing GPCR heteromers in human post-mortem brains with high sensitivity.

## 1. Introduction

G-protein coupled receptor (GPCR) oligomerization is an important phenomenon in which receptors form complexes displaying singular characteristics. Thus, these kinds of receptor–receptor interactions lead to relevant effects in receptors’ functioning, such as biosynthesis, plasma membrane diffusion or pharmacology (for review see [1]). For instance, the formation of receptor complexes is related to mechanisms such as functional selectivity or biased signaling, in which ligands can preferentially trigger the activation of concrete signaling pathways that can ultimately lead to better benefit/risk profiles (for review see [2,3,4]). In line with this, we recently described that the biased activity of a functionally selective antipsychotic ligand was dependent on the oligomerization of dopamine D_2_ and adenosine A_2A_ receptors (D_2_R and A_2A_R, respectively) in the mouse striatum [5]. 

The existence of direct receptor–receptor interactions, namely, oligomerization, between D_2_R and A_2A_R has been widely studied, particularly within the context of Parkinson’s disease (PD), the second most common neurodegenerative disorder [6,7]. The existence of D_2_R/A_2A_R oligomers in the striatum, where the selective dopaminergic denervation occurs in PD, has been postulated since the beginning [8]. However, the unambiguous demonstration that D_2_R/A_2A_R oligomers exist in native tissue was recently confirmed by means of proximity ligation assay, immunoelectron microscopy and ligand fluorescence resonance energy transfer (FRET)-based approaches in mouse [9,10,11] and monkey [10] striatum. In addition, it was possible to elucidate that the D_2_R/A_2A_R oligomerization state was reduced in the striatum from 6-hydroxydopamine (6-OHDA) lesioned rodents (a model for PD). Conversely, we did not manage to robustly assess the presence of D_2_R/A_2A_R complexes in human post-mortem brains from healthy and PD subjects. Thus, although other authors have successfully used similar approaches to detect protein–protein interactions (i.e., dopamine transporter/α-synuclein) in human necropsies [12], we obtained a very low signal-to-background (S/B) ratio, limiting the assessment of oligomerization. The nature of the necropsies, together with the variable time and conservation protocols of tissue extraction, could explain the difficulties to obtain robust results. 

**Figure 1:**
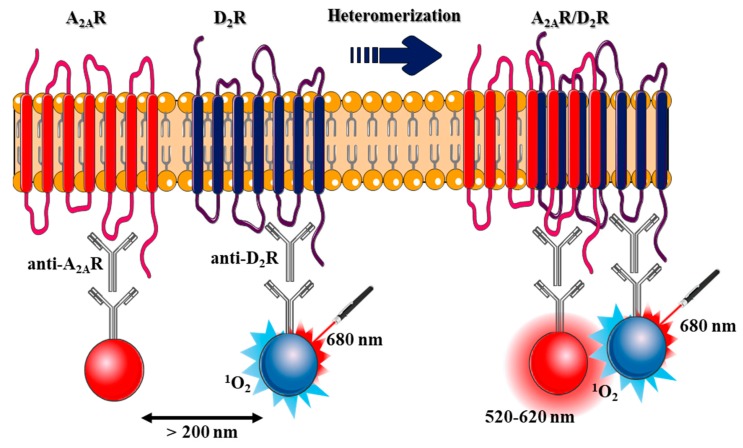
AlphaScreen design. Illustration of the specific AlphaScreen protein–protein interaction assay designed for A_2A_R/D_2_R heteromer assessment in native tissue. Anti-goat coated acceptor beads (red spheres) were generated to capture a goat anti-A_2A_R antibody bound to the receptor within the membrane extract. Anti-rabbit coated donor beads (blue spheres) capture the immune complex between the rabbit anti-D_2_R antibody and the receptor again within a membrane extract. A_2A_R/D_2_R heteromerization brings donor beads into close proximity (< 200 nm) to the acceptor beads. The excitation of the donor beads at 680 nm generates singlet oxygen (^1^O_2_) molecules triggering a chemical reaction within the acceptor beads, which results in a sharp peak of fluorescent emission at 520–620 nm. Figure designed using image templates from Servier Medical Art https://smart.servier.com/image-set-download/.

Here, we propose a novel approach based on the use of the AlphaSrceen technology. This technology, in which Alpha is the acronym for Amplified Luminescent Proximity Homogeneous Assay, is a wash-free assay based on LOCI™ (Luminescent Oxygen Channeling Immunoassay) technology [13,14]. In brief, this tool consists of the use of two types of latex nanobeads (200 nm diameter): (i) donor beads loaded with a photosensitizer (phthalocyanine); and (ii) acceptor hydrogel-coated beads containing a chromophore (i.e., rubrene, europium). The proximity between beads is detected through the excitation of the donor bead with 680 nm light, which leads to the production of a singlet oxygen, and the concomitant chemiluminescence from the acceptor bead (i.e., rubrene) upon exposure to singlet oxygen (Figure 1) [15]. The Alpha technology has been successfully used to detect protein–protein interactions and has been shown to display a high sensitivity and a very high S/B ratio, which may be suitable for implementing high-throughput screening (HTS) assays [16]. Of note, in the present study, we also calculated the Z’ value, which is a statistical parameter that allows determining assay robustness [17]. The Z’ factor takes into account both the S/B and CV%; thus, it is a powerful indicator of how reliable and reproducible a named assay is. Accordingly, we designed a similar approach to that used in proximity ligation assay, in which antibodies against D_2_R and A_2A_R were selectively labelled with donor and acceptor beads (see methods) to engage in a singlet oxygen energy transfer dependent on the formation of D_2_R/A_2A_R oligomers. Importantly, we first aimed to validate results previously obtained in animal models and, subsequently, we evaluated the oligomerization state in healthy and PD patients.

## 2. Results

### 2.1. Design and Validation of the AlphaScreen Assay

The first aim of this work was to engineer a sensitive AlphaScreen-based assay to assess the presence of D_2_R/A_2A_R complexes in native tissue. In this assay, specific primary antibodies recognize the receptors of interest, which in turn can be recognized by secondary antibodies labelled with donor and acceptor beads able to engage in a singlet oxygen energy transfer (Figure 1). 

**Figure 2:**
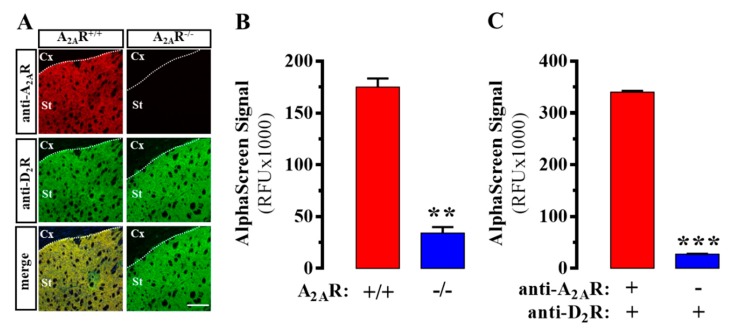
A_2A_R/D_2_R heteromer assessment in mouse striatum and human caudate using AlphaScreen. (**A**) Representative images of A_2A_R and D_2_R immunoreactivities in the dorsal striatum of wild-type (A_2A_R^+/+^) and A_2A_R^-/-^ CD-1 mice. Cx, cortex; St; striatum. (see Methods). Scale bar: 350 µm. (**B**) AlphaScreen signal obtained in striatal slices from A_2A_R^+/+^ and A_2A_R^-/-^ CD-1 mice (see Methods). (**C**) AlphaScreen signal obtained in human caudate, either with or without using the two primary antibodies (see Methods). Results are presented as mean ± SEM of three independent experiments performed in triplicate. ** *p* < 0.01, *** *p* < 0.001, Student’s *t*-test.

First, we validated the specificity of the antibodies used against D_2_R and A_2A_R. We consistently detected both receptors in mouse striatum, thus demonstrating their co-distribution within this brain area (Figure 2A), as previously described [11]. As expected, A_2A_R immunoreactivity was not observed in A_2A_R deficient (A_2A_R^-/-^) mice (Figure 2A). Subsequently, we aimed to assess the existence of D_2_R/A_2A_R oligomers in striatal membranes from mice and human post-mortem brains using a homemade AlphaScreen assay. We first gauged the specificity of the assay comparing the signal of wild-type (i.e., A_2A_R^+/+^) and A_2A_R^-/-^ mice. A robust AlphaScreen signal was observed in A_2A_R^+/+^ mice, which was significantly (P = 0.0012) higher than that observed in A_2A_R^-/-^ mice (Figure 2B). Of note, we took advantage of A_2A_R^-/-^ mice to validate results, but other approaches could be used (i.e., interfering oligomer formation), as previously described [18]. We calculated the Z’ value, which, as mentioned above, is a statistical parameter determining assay robustness [17]. Importantly, an assay is considered excellent when the Z’ value ranges between 0.5 and 1, while values lower than 0.5 are regarded as marginal [17]. Interestingly, the AlphaScreen signal obtained in mice striatal membranes provided a Z’ = 0.52, thus confirming the validity of the assay. On the other hand, when the S/B ratio was determined, a value of 4.6 was observed. As with the Z’ value, there is an agreement in which S/B ratios around 10:1 are desirable, although lower values are acceptable when the dispersion of the measurements is low [17]. Subsequently, we aimed to reveal the existence of D_2_R/A_2A_R oligomers in human caudate through the same experimental approach. As the negative control, caudate membranes were incubated with all the components of the assay, excepting one of the primary antibodies, specifically, the goat anti-A_2A_R antibody (Figure 1). Similar to the results obtained in mice, a robust AlphaScreen signal was observed in human caudate membranes, which was significantly (P < 0.0001) higher than that observed in the negative control (Figure 2C). Notably, a better Z’ value (0.94) and S/B ratio (12.5) were obtained, compared to those determined in mouse samples. Overall, we confirmed for the first time the existence of D_2_R/A_2A_R oligomers in human caudate through a new AlphaScreen approach.

### 2.2. Evaluation of D_2_R/A_2A_R Oligomerization in PD Patients

**Figure 3:**
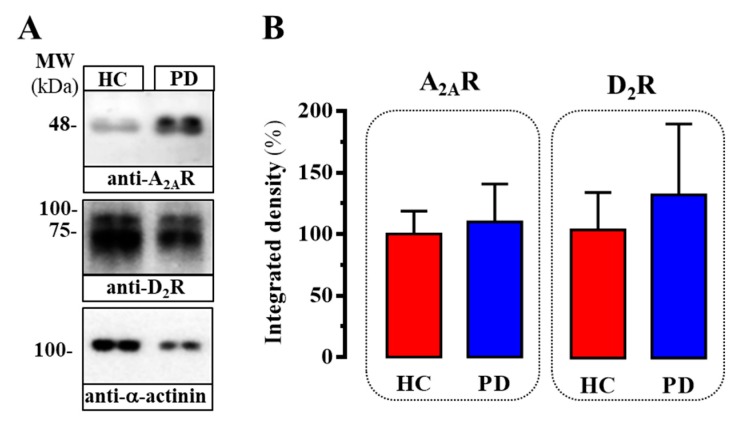
Expression of A_2A_R and D_2_R in caudate from PD subjects. (**A**) Immunoblot showing the expression of A_2A_R and D_2_R in caudate membranes from HC and PD subjects. Caudate membranes were analyzed by SDS-PAGE (60 μg of protein/lane) and immunoblotted using goat anti-A_2A_R, rabbit anti-D_2_R and rabbit anti-α-actinin antibodies (see Methods). (**B**) Relative quantification of A_2A_R and D_2_R expression. The immunoblot protein bands corresponding to A_2A_R, D_2_R and α-actinin from control (*n* = 6) and PD (*n* = 6) individuals were quantified by densitometric scanning. Values were normalized by the respective amount α-actinin in each lane to correct for protein loading. Results are expressed as percentage (mean ± SEM) of the control.

Once the quality of the new AlphaScreen assay to assess D_2_R/A_2A_R oligomers in human tissue was validated (i.e., Z’ factor and S/B), we aimed to gauge potential changes associated with PD pathophysiology. To this end, we first analyzed the expression of D_2_R and A_2A_R in human caudate membranes by immunoblot. Thus, D_2_R and A_2A_R expression was ascertained by the presence of a protein band of molecular weight ~80 kDa and ~45 kDa, respectively (Figure 3A), as previously demonstrated [19]. Interestingly, when the relative density of both receptors was quantified in healthy control (HC) and PD subjects, no significant differences were observed (Figure 3B). Regardless, no changes in the relative expression of the receptors were observed, we aimed to determine the abundance of D_2_R/A_2A_R oligomers in HC and PD subjects. 

**Figure 4:**
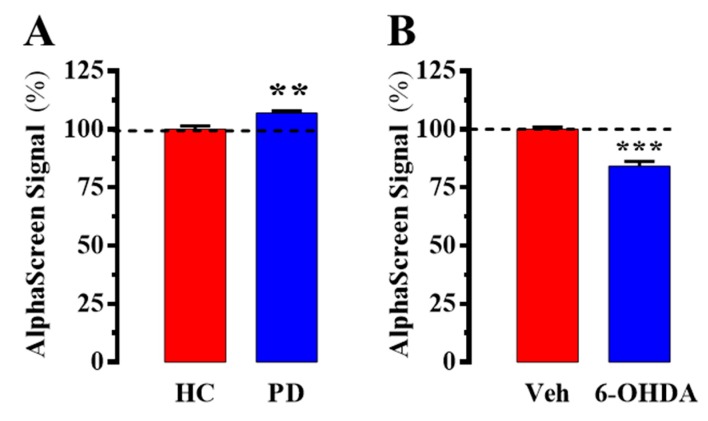
AplhaScreen A_2A_R/D_2_R heteromer assessment in a PD animal model and PD post-mortem brains. (**A**) AlphaScreen signal obtained in human caudate from healthy control (HC) and PD subjects. (**B**) AlphaScreen signal obtained in striatal membranes from vehicle (Veh) and 6-OHDA-lesioned rats (see Methods). Results are presented as mean ± SEM of three independent experiments performed in triplicate. The number of samples was n = 6 for each condition. The dotted line indicates the 100 % of the signal, assigned to that obtained in HC or Veh conditions, respectively. ** *p* < 0.01; *** *p* < 0.001, Student’s *t*-test.

As shown in Figure 4A, a slight but significant increase (~7%; P = 0.003) was observed in caudate from PD subjects, thus indicating that despite no significant changes in total density D_2_R and A_2A_R had a higher interaction index. It is important to note that these results were opposed to those observed in PD animal models [9,10,11]. Therefore, we decided to assess D_2_R/A_2A_R oligomerization in the 6-OHDA hemiparkinsonian rat model by means of the AlphaScreen assay. To this end, striatal membranes of 6-OHDA-lesioned rats were prepared and incubated with the primary and secondary (labelled with donor and acceptor beads) antibodies. As previously described, the 6-OHDA-lesioned striatal hemisphere showed a significant decrease (~16.4%; P = 0.0005) of D_2_R/A_2A_R oligomers when compared to that observed in the contralateral saline-treated striatal hemisphere (Figure 4B). One possible explanation would be that some differences exist between PD in humans and in animal models of parkinsonism (i.e., 6-OHDA-lesioned rat), but further work is needed to ascertain these divergences. Nevertheless, our study strongly supports the need for developing novel techniques able to study human samples overcoming the highly valuable but also limited information obtained in animal models.

## 3. Discussion

Over the last years, several new pharmacological concepts (i.e., biased signaling and allosteric drug function) have emerged as putative mechanisms behind GPCR drug effect divergences [2,3,4]. GPCR oligomerization constitutes a key phenomenon in GPCR biology that may contribute to the occurrence of such pharmacological mechanisms. Hence, upon direct receptor–receptor interactions, a new pharmacological entity may be shaped with a genuine pharmacological profile eventually different from that shown by the single protomers [2,3,4]. Indeed, the existence of GPCR oligomers and its likely association to pathological conditions constitute a major contention in pharmacology, nowadays [20]. D_2_R/A_2A_R oligomerization has been extensively studied in the context of PD [21]. However, although relevant data has been gained based on experimentation with animal models (i.e., the 6-OHDA hemiparkinsonian rat), the unambiguous demonstration of D_2_R/A_2A_R oligomers in human post-mortem brains has not been achieved. To our knowledge, here we were able to assess for the first time the existence of D_2_R/A_2A_R oligomers in human caudate. Moreover, we further preliminarily confirmed that D_2_R/A_2A_R oligomers may be increased in PD patients when compared to healthy subjects. Overall, this notion may open a novel way to finely characterize the pathological fingerprint related to GPCR oligomerization.

The main objective of the present work consisted of developing a new AlphaScreen-based assay to disclose the existence of GPCR oligomers in human post-mortem brains. In line with this, we aimed to characterize the quality of such assay by determining two critical parameters while implementing HTS assays. Firstly, we determined the S/B, which makes it possible to establish the dynamic range of the assay [17,22]. Interestingly, in human samples, this parameter achieved best-fit values, being higher than 10:1 (i.e., 12.5); conversely, although in some assays it may be considered acceptable, in mouse samples, the S/B was lower than 10:1 (4.5). The capability of the selected antibodies to recognize the mice and human species could be the main explanation for this difference, but other factors could also be relevant. Similarly, the Z’ value, which makes it possible to ensure that the assay is tightly replicable, achieved the best-fit values (1 > Z’ > 0.5), especially in the case of human samples (i.e., 0.94). Altogether, the results obtained supported the validity of the new AlphaScreen assay for assessing GPCR oligomerization in human brain tissue.

Next, we aimed to ascertain changes in D_2_R/A_2A_R oligomerization status under pathological conditions. Of note, we observed a slight (~7%) but significant increase of D_2_R/A_2A_R oligomers in human samples. Needless to say, our assay was a pilot study in which only a reduced number of PD post-mortem brains (*n* = 6) were analyzed; thus, future work will be needed to establish further associations between D_2_R/A_2A_R oligomer density and PD pathophysiology. Nevertheless, this result was unexpected, since all data in the literature obtained in PD animal models support a decrease in D_2_R/A_2A_R oligomerization [9,10,11]. Accordingly, we validated previous results using the AlphaScreen assay in the 6-OHDA hemiparkinsonian rat, which reinforced the results observed in human post-mortem brains. On the other hand, these results pointed to some divergences between human and animal samples. We cannot rule out that some differences exist between PD in humans and in animal models, or that PD medications, which are not applied in animal models, might have an effect in D_2_R/A_2A_R oligomerization. In line with this, the clinical concept of personalized treatments in PD relies on the genomic profile of patients [23,24], which is also important for predicting the occurrence of drug-induced side effects (i.e., l-Dopa-induced dyskinesia) [25]. Altogether, our data strongly support the need for novel techniques permitting a better characterization of the molecular changes occurring in human diseases; thus, although animal models provide important and relevant data, further confirmation is needed in human tissue.

Finally, we also determined the relative D_2_R and A_2A_R density in caudate from healthy and PD subjects. We observed a slight but not significant increase in D_2_R and A_2A_R expression in PD patients, compared to that observed in HC. The relative D_2_R and A_2A_R density in post-mortem brains from PD patients is a matter of debate, since both up- and down-regulation have been previously reported. Accordingly, some reports have indicated that D_2_R is upregulated [26], downregulated [27] or unaltered [28,29,30] in caudate nucleus, nucleus accumbens and putamen from PD brains. Similarly, the adenosinergic system has also been shown to be dysregulated in PD [31], and a significant increase in A_2A_R density has been found in postmortem caudate-putamen from PD subjects [29,32]. Nevertheless, the relevant finding of the present assay was that irrespective of possible changes in receptors’ expression, a higher interaction between D_2_R and A_2A_R would occur, which might be considered both as a pathological fingerprint and a pharmacological target in PD.

## 4. Materials and Methods

### 4.1. Human Brain Samples

Brain tissue was obtained from the Institute of Neuropathology Brain Bank following the guidelines of Spanish legislation on this matter and the approval of the local ethics committee. The left cerebral and cerebellar hemispheres were immediately cut into 1-cm-thick coronal sections, and the caudate was rapidly dissected, frozen on metal plates over dry ice, placed in individual air-tight plastic bags, numbered with water-resistant ink, and stored at −80 °C until use for biochemical studies. Mean age, post-mortem interval (PMI), and brain pH did not differ significantly between healthy controls and PD subjects (Table 1). PD subjects were treated with pro-dopaminergic drugs (i.e., l-DOPA), and they did not show cognitive deficits. Thus, while four pathological cases were from early PD subjects (an average of 3.5 years of diagnosis) without dyskinesia, the other two were from late PD. Neuropathologic diagnosis was done following the Braak staging of brain pathology related to sporadic Parkinson’s disease [33]. Thus, Lewy body disease (LBD) and Braak stage 4–5 was confirmed in all PD necropsies. Of note, cases with combined pathologies, including hippocampal sclerosis, α-synucleinopathies, other tauopathies (particularly argyrophilic grain disease), TDP-43 proteinopathies, vascular diseases, infectious and autoimmune diseases, and cases with metabolic syndrome were excluded from the present study. Only cases with concomitant mild small vascular disease were accepted. Cases with evident long agonic state, hypoxia and seizures were excluded.

### 4.2. Animals

Wild type and A_2A_R^-/-^ [34] CD-1 male and female mice weighing 25–50 g were used at 2–3 months of age. Also, Sprague-Dawley male and female rats (Charles River Laboratories, L’Arbresle, France) weighing 200–250 g were used at 2–3 months of age. The University of Barcelona Committee on Animal Use and Care (CEEA-UB) approved the protocol (Code 10033, 04 February 2018). Animals were housed and tested in compliance with the guidelines described in the Guide for the Care and Use of Laboratory Animals [35] and following the European Union directives (2010/63/EU), FELASA and ARRIVE guidelines. All efforts were made to minimize animal suffering, and the number of animals used. Animals were housed in groups of five in standard cages with ad-libitum access to food and water and maintained under 12 h dark/light cycle (starting at 7:30 AM), 22 °C temperature, and 66% humidity (standard conditions).

### 4.3. Surgery

Experimental hemiparkinsonism was induced in rats by means of a unilateral lesion of the medial forebrain bundle, which was destroyed using a 6-OHDA injection. In brief, rats were anesthetized with a ketamine/xylazine combination (75 mg/kg ketamine hydrocloride/10 mg/kg xylazine hydrochloride, intraperitoneally; Sigma-Aldrich, St. Louis, MO, USA) and immobilized in an adapted digital lab stereotaxic device (Stoelting Co., Wood Dale, IL, USA). Also, rats were treated with desipramine hycrochloride (10 mg/kg; Sigma-Aldrich) 30 min before 6-OHDA injection. An incision (0.5 cm) was performed in the skin of the skull to unilaterally lesion the right striatum with 6-OHDA (8 μg of 6-OHDA in 4 μL of saline solution containing 0.05% ascorbic acid; Sigma-Aldrich) by means of a Hamilton syringe (model 701, Reno, NV, USA). The stereotaxic coordinates, following the atlas of the rat brain, were respect to bregma: AP = −2.2 mm, ML = −1.5 mm and DV = −7.8 mm. The 6-OHDA solution (or saline as a negative control; sham) was injected manually at a rate of 1 μL/min, and after the injection, the needle was left in place for 5 min before slowly retracting it to prevent reflux. Rats were then quickly warmed and returned to their cages. Finally, two weeks after the lesion, the extent of dopamine deafferentation was validated by assessing the rotating behavioral response to l-DOPA (3,4-Dihydroxy-l-phenylalanine; Abcam Biochemicals, Cambridge, UK) administration. In brief, rats received an intraperitoneal injection of l-DOPA (50 mg/kg) in the presence of benserazide hydrochloride (25 mg/Kg i.p; Sigma-Aldrich) and the number of full contralateral turns counted during a 2 h period. Dopamine deafferentation was considered successful in those animals that made at least 200 net contralateral rotations.

### 4.4. Membrane Preparation

Mice and rat striatum or human caudate samples were homogenized in ice-cold 10 mM Tris HCl, pH 7.4, 1 mM EDTA, 300 mM KCl buffer containing a protease inhibitor cocktail (Roche Molecular Systems, Belmont, CA, USA) using a Polytron for three periods of 10 s each. The homogenate was centrifuged for 10 min at 1000× g. The resulting supernatant was centrifuged for 30 min at 12,000× g. The membranes were dispersed in 50 mM Tris HCl (pH 7.4) and 10 mM MgCl_2_, washed, and resuspended in the same medium as described previously [36]. Protein concentration was determined using the BCA protein assay kit (Thermo Fisher Scientific, Inc., Rockford, IL, USA) and 60 μg of protein was used for immunoblotting.

### 4.5. Gel Electrophoresis and Immunoblotting

Sodium dodecyl sulfate polyacrylamide gel electrophoresis (SDS/PAGE) was performed using 10% polyacrylamide gels. Proteins were transferred to PVDF membranes using a semidry transfer system and immunoblotted with the indicated primary antibody (see below; AlphaScreen assay) and then HRP-conjugated goat anti-guinea pig (1:10,000) or goat anti-rabbit IgG (1:30,000). The immunoreactive bands were visualized using a chemiluminescent detection kit (Pierce Biotechnology, Rockford, IL, USA) and an Amersham Imager 600 (GE Healthcare Europe GmbH, Barcelona, Spain).

### 4.6. AlphaScreen Assay

Human caudate, and mouse and rat striatal membranes were homogenized in Alpha immunoassay buffer (AL001C; PerkinElmer, Waltham, MA, EEUU) and protein concentration determined. Subsequently, 10 μL (10 μg of protein/well) was placed in white 384-well plates (384 Well Small Volume™ HiBase Microplates, Greiner Bio-one, Kremsmünster, Austria). Next, membranes were incubated with 3 nM donor primary antibody (rabbit anti-D_2_R; AB_2571596, Frontier Institute Co. Ltd., Shinko-nishi, Ishikari, Hokkaido, Japan) plus 10 nM acceptor primary antibody (goat anti-A_2A_R; sc-7504, Santa Cruz Biotechnology, Dallas, TX, USA) to a final volume of 20 μL overnight at 4 °C. As a negative control, samples were also incubated in the absence of acceptor primary antibody. Also, a background signal was determined in wells where all reagents were added without sample. Pipetting up and down before incubation was performed to mix all the reagents. After the overnight incubation, plates were tempered at 22 °C and 5 μL donor (anti-rabbit IgG donor beads, AS105D, Perkin Elmer) and 5 μL acceptor beads (anti-goat IgG acceptor beads, AL107, Perkin Elmer) were added (20 μg/mL). After 1 h incubation in the dark at room temperature, the plate was read using a CLARIOstar plate-reader (BMG Labtech, Durham, NC, USA). 

### 4.7. Statistics

Data are represented as mean ± standard error of mean (SEM). The number of samples/animals (n) in each experimental condition is indicated in the corresponding figure legend. Comparisons among experimental groups were performed by Student’s *t* test, using GraphPad Prism 6.01 (San Diego, CA, USA), as indicated. Statistical difference was accepted when *P* < 0.05. The Z’ value was calculated according to the following formulae [17]: Z’= 1 – [(3SD of sample + 3SD of control) / (mean of sample – mean of control)]. 

## 5. Conclusions

In summary, we have designed and validated a new AlphaScreen assay that was used to disclose for the first-time the existence of GPCR heteromers in human post-mortem brain. Specifically, we have outlined that the D_2_R/A_2A_R oligomerization status may be increased in the caudate from PD patients. This fact may help to better understand the disease etiology and to design selective combined pharmacotherapeutic strategies, restoring the unbalanced D_2_R/A_2A_R heteromer function potentially associated with PD.

## Figures and Tables

**Table 1:** Postmortem brain samples’ characteristics.

Parkinson’s Disease Subjects (*n* = 6) and Matched Controls (*n* = 6)				
Group	Gender (M/F)	Age (years)	PMI (h)	pH
**PD**	4M/2F	71.3 ± 13.1	6.8 ± 3.1	6.3 ± 0.1
**HC**	4M/2F	72.5 ± 9.2	5.1 ± 1.5	6.2 ± 0.1

Group values are means ± SEM. PD: Parkinson’s disease, HC: healthy control, F: female, M: Male, PMI: Postmortem Interval.

## Abbreviations

GPCR	G protein-coupled receptor
D_2_R	Dopamine D_2_ receptor
A_2A_R	Adenosine A_2A_ receptor
HC	Healthy control
PD	Parkinson’s disease
6-OHDA	6-hydroxydopamine
S/B	Signal-to-background
HTS	High throughput screening
LBD	Lewy body disease
l-DOPA	3,4-Dihydroxy-l-phenylalanine
BCA	Bicinchoninic acid assay
SDS/PAGE	Sodium dodecyl sulfate/polyacrylamide gel electrophoresis
